# Minimum acceptable diet and associated factors among children aged 6–23 months in Jig-Jiga, Somali region, eastern Ethiopia, 2022

**DOI:** 10.1186/s40795-023-00740-x

**Published:** 2024-01-11

**Authors:** Shukri Farah, Tariku Derese, Legesse Abera

**Affiliations:** 1 Department of Public Health, College of Medicine and Health Sciences, Dire Dawa University, Dire Dawa, Ethiopia; 2 Health Professional in Somalia Regional State Administration, Jijiga, Ethiopia

**Keywords:** Minimum acceptable diet, Minimum dietary diversity, Minimum meal frequency, Eastern Ethiopia, Jig-Jiga

## Abstract

**Background:**

The minimum acceptable diet is the proportion of children aged 6–23 months who consumed the minimum meal frequency and minimum dietary diversity during the previous day or night. The minimum acceptable diet assesses both micronutrient adequacy and the quantity of food consumed during the previous day or night. Inappropriate infant and young child feeding practices during this period result in significant threats to child health through impaired cognitive development. Therefore, this study aims to assess the minimum acceptable diet and associated factors among children aged 6–23 months in Jig-Jiga, Somali region, Eastern Ethiopia.

**Methods:**

A community-based, cross-sectional study was conducted in Jig-Jiga town. A systematic random sampling technique was used to select 536 children aged 6–23 months with their mothers. Data were collected using a pre-tested, structured questionnaire. The data were entered into Epi-data 3.1. The data were cleaned and analyzed using SPSS v20. Bi-variable and multivariable logistic regression analyses were done, and model fitness was checked and tested by the Hosmer-Lemeshow goodness of fit test. The results of the adjusted odds ratio with 95% confidence intervals and P < 0.05 were considered statistically significant.

**Result:**

The overall prevalence of a minimum acceptable diet was 47.2% (95% CI: 43.1–51.6). Occupation of fathers (AOR = 0.5, 95%CI: 0.3–0.8), child age of 6–11 months (AOR = 3.6, 95%CI: 1.7–7.7), age of the mother 15–24 years (AOR = 7.6, 95%CI: 1.5–38.146), 25–34 years (AOR = 5.56, 95%CI: 1.17–26.325), mothers who had only one under-five child (AOR = 2.1, 95%CI: 1.298–3.471), and media as a source of information (AOR = 0.16, 95%CI: 0.061–0.433) were associated with the minimum acceptable diet.

**Conclusion:**

This study showed that the prevalence of a minimum acceptable diet was low. Factors associated with a minimum acceptable diet included the father’s occupation, the child’s age, the mother’s age, having one under-five child, and the media as a source of information. Therefore, interventional strategies that focus on family planning and advocacy for infant and young child feeding are required to improve the provision of a minimum acceptable diet.

## Background

Proper nutrition from onset to 24 months of age is a critical window of opportunity that determines a child’s well-being. The introduction of appropriate nutrition at age 6 months, together with sustaining breastfeeding until 2 years of age, warrants optimal growth and development and maintains a healthy life throughout the life cycle [1, 2].

Minimum dietary diversity is used to measure the quality of an infant’s or young child’s complementary diet, while minimum meal frequency is used as a proxy measure of energy intake. Minimum acceptable diets measure multiple dimensions of infants’ and young children’s diets; those children aged 6–23 months who meet both macronutrient and micronutrient requirements [3, 4].

In many countries, less than a quarter of children are reported to not be getting the nutrition they need to grow well, particularly in the crucial first 1000 days [5–7]. Consumption of acceptable dietary standards has numerous benefits, including enhanced linear growth, better cognitive development, and high school achievement, reduced risk of non-communicable disease, increased body immunity systems, and increased productivity during adult life [3, 8, 9].

The World Health Organization recommends the following indicators: proper complementary feeding: starting solids, semi-solids, or soft foods; minimum meal frequency; minimum dietary diversity; a minimum acceptable diet; and consumption of iron-rich or iron-fortified foods [10].

“The first 1000 days” is critical to encourage and support appropriate complementary feeding for children aged 6–23 months. It is estimated that adequate breastfeeding and complementary feeding prevent 13% and 6% of malnutrition in under-five children, respectively [11]. Good infant and young child feeding practices promote normal growth of the child and better health, mental, and physical development [12]. Inappropriate complementary feeding of 6- to 23-month-old children results in significant threats to child health through impaired cognitive development, compromised educational development, and low economic productivity, which become difficult to reverse in their life cycle [13–15]. Inadequate amounts and quality of complementary foods, poor child feeding practices, and high rates of infection harm health and growth in children younger than 2 years of age [16].

Studies in different areas showed that consumption of the recommended minimum acceptable diet (MAD) in children aged 6–23 months greatly varied from area to area, with the lowest proportions of minimum acceptable diet reported in Nepal and the Philippines, respectively, at 8% and 6.7% [17, 18]. Sub-Saharan African countries carry the highest risk of the minimum acceptable diet practice of the global burden. In many low-income countries, including Ethiopia, meeting minimum dietary diversity (MDD) and minimum meal frequency (MMF) standards has been a serious challenge, especially in areas where household food security is poor [15].

In Ethiopia, only 8% of infants aged 6–23 months receive complementary foods while continuing to be breastfed. The prevalence of minimum dietary diversity (MDD), minimum meal frequency (MMF), and minimum acceptable diet (MAD) in Ethiopia was 14%, 45%, and 7%, respectively [19, 20]. Chronic malnutrition among children continues to be a major public health issue in Ethiopia because of poor minimum acceptable diet practice. Despite the success made in recent decades with a reduction in under-five child mortality, about 38% of children under the age of five were stunted in 2016 [21].

Ethiopia has implemented a national nutrition program, and the nutrition indicators were integrated into the nation’s 5-year growth and transformation plan to finalize and eliminate any form of malnutrition in Ethiopia by 2030. Even though there are a lot of nutrition intervention programs in Ethiopia, suboptimal feeding practices are still prevalent, and the minimum acceptable diet (MAD) has only risen from 3 to 7% over the past 11 years (2005–2016) [22].

Information on dietary diversity, meal frequency, the minimum acceptable diet, and associated factors is needed to prioritize, design, and initiate further intervention programs aimed at improving dietary diversity and meal frequency to reduce undernutrition in children. However, there is a lack of evidence about the minimum acceptable diet in Somalia’s regional state, and this is the first study in this area. Additionally, most of the previous studies conducted in the countries were about child dietary diversity practices and missed meal frequency, which is a very important measure of energy intake. Therefore, the aim of this study was to assess the minimum acceptable diet and associated factors among children aged 6–23 months in Jigjiga town, Somali region, Eastern Ethiopia.

## Methods and materials

### Study area and study period

**Fig. 1:**
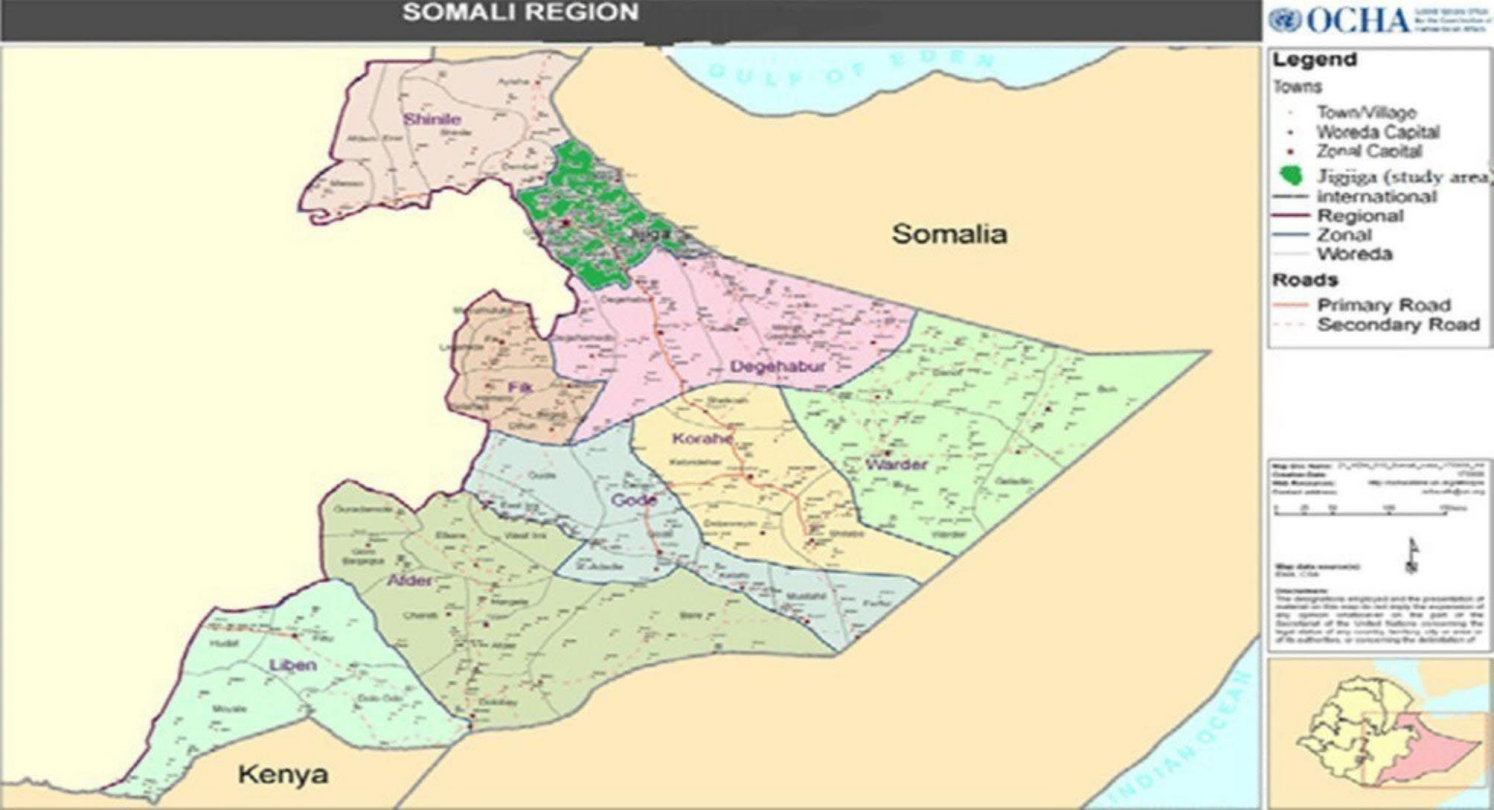
Map of the study area with green, Jig-Jiga town, Somali region, Eastern Ethiopia, 2022

Jig-jiga is located in Eastern Ethiopia, which is 632 km away from Addis Ababa and 105 km from the ancient walled city of Harar. Based on figures from the central statistical agency in 2007, Jig-Jiga has an estimated total population of 277,560 (the annual growth rate is 2.6%), of whom 46.7% are females and 53.3% are males. Among the total population, 47% are under 15 years old, whereas 3.2% are under the age of 1, estimated to be 55.4 years for females and 58.7 years for males. The reproductive age group is 22.8%, and the total fertility rate (TFR) is 6.6. Life expectancy at birth is estimated to be 55.4 years for females and 58.7 years for males [23]. The study area map is shown with a green line (Fig. 1). The study was conducted in Jig-Jiga town, Somali region, from October 7 to November 2, 2022.

### Study design

A community-based cross-sectional study design was conducted among children aged 6–23 months with mothers or caregivers living in Jig-Jiga town.

### Source and study population

All children aged 6 to 23 months who were living in Jig-Jiga town were sources of population. All the randomly selected children aged 6 to 23 months who were living in Jig-Jiga town were part of the study population.

### Eligibility criteria

All children aged 6–23 months who lived in Jig-Jiga town for at least 6 months were included in the study. Children whose mothers or caregivers were absent from the household or unable to respond because of the child’s illness or their illness at the time of data collection were excluded from the study.

### Sample size and sampling procedure

A sample size was calculated for a minimum acceptable diet among children aged 6–23 months by considering the following assumptions: a two-sided confident interval of 95% and a power of 80%, the sample size was calculated using Epi Info version 7 software programs, and the maximum sample size was taken for the final required sample size ratio: 1 to 1, with ANC visits exposed at 58% and none exposed at 42% [3].

Finally, the required sample size for this particular study was determined by taking the maximum sample, which was 340. Adding 1.5 of the design effect [3] and 5% of the non-response rate, the final sample size was 536. Jig-jiga town has 20 kebeles, out of which 6 kebeles were selected by simple random sampling (kebele 17, kebele 04, kebele 14, kebele 09, kebele 12, and kebele 07), and the total number of mothers with children aged 6–23 months was obtained from those 6 kebeles. Then the calculated sample size was proportionally allocated to those selected kebeles. Lastly, study participants were selected by systematic random sampling using the interval “K,“, where K = N/n = 7200/386 = 19.

### Operational definitions

Exclusive breastfeeding means giving only breast milk to infants up to 6 months old and allowing only ORS drops, syrups, or medicines. Exclusive breastfeeding (EBF) is the practice of feeding breast milk during the first 6 months and no other liquids or solid foods, except medications [24].

Minimum Acceptable Diet: “proportion of children 6–23 months of age who had at least the minimum dietary diversity and minimum meal frequency during the previous day” [25].

Minimum Dietary Diversity: “Proportion of children aged 6–23 months who receive five or more food groups out of the eight food groups in the last 24 hours, for breastfeeding children five food groups out of eight foods, and for non-breastfeeding children four food groups out of seven foods, and a minimum of 2 cups of milk consumption” [26].

Minimum Meal Frequency: “proportion of breastfeeding and non-breastfeeding children aged 6–23 months who receive soft, solid, and semi-solid meals in the last 24 hours.“ Breastfeed infants aged 6–8 months 2 times in the last 24 hours; breastfeed infants and young children aged 9–23 months 3 times in the last 24 hours; and non-breastfeed infants and young children aged 6–23 months at least 4 times in the last 24 hours” [27].

Postnatal care: the WHO stated that “postnatal care” (PNC) is defined as care given to the mother and her newborn baby immediately after the birth of the placenta and for the first 42 days of life [28].

Antenatal care (ANC) is a maternal health care service that’s provided throughout pregnancy to ensure the best possible health outcome for both the mother and the newborn [29].

### Data quality control and procedure

Data were collected using a pre-tested, structured, and interview-administered questionnaire adapted from previous studies (3, 20, 26, 30, and 31). The questionnaires have four parts: socio-demographic factors, household-related factors, child-related factors, and obstetric-related factors. Minimum dietary diversity was collected as a simple count of 8 food groups consumed by the child in 6–23 months for those children who were breastfed (5 food groups out of 8) and non-breastfed children (4 food groups out of 7) before the data collection to calculate minimum dietary diversity and minimum meal frequency over the past 24 h by using WHO guidance [32]. The minimum acceptable diet was calculated based on the proportion of children 6–23 months of age who had at least the minimum dietary diversity and minimum meal frequency during the previous day. The proportion of children 6–23 months of age who had at least the minimum dietary diversity and minimum meal frequency during the previous day. Eight data collectors (six diplomas and a BSC nurse) who have experience in data collection and supervision collected the data after two days of training. The authors had no access to information that could identify individual participants during or after data collection. On the spot, supervisors checked and reviewed all the completed questionnaires to ensure the completeness and consistency of the information collected, and any incorrectly filled or missed questionnaires were given back to the respective data collectors for correction.

### Data processing and analysis

First, the data were checked manually for completeness and consistency. Data were checked, cleaned, coded, and entered into EPI 3.1, then exported to SPSS version 20 for statistical analysis. Descriptive analysis using frequencies, tables, and charts was used for summarizing the data based on relevant variables. Binary logistic regression was applied to see the association between the minimum acceptable diet and other independent variables. Variables with a p-value of 0.25 [26] were candidates for multivariable regression. In a multivariable logistic regression model, fitness was checked and tested by the Hosmer-Lemeshow goodness of fit test. The odds ratio (OR) along with the 95% confidence interval (CI) were used to measure the strength of association between variables, and the level of statistical significance was declared at p-values < 0.05.

## Result

A total of 536 mother-child pairs participated in the study, which had a response rate of 100%. More than half of the mothers and caregivers in this study (293, 54%) were between the ages of 25 and 34 years. The majority (502, or 93.7%) of mothers were married; 499, or 93%, were Muslims; and 6.9% of them were of another religion. Nearly half (259, 48.3%) and 415, 77.4%, of the mothers and the husbands were in secondary and above, respectively. About 253 (47.2%) of children were in the age range of 6–11 months, and 299 (55.8%) and 237 (44.2%) children in this study were females and males, respectively (Table 1).

**Table 1:** Socio-demographic characteristics of the study participants, in the study of minimum acceptable diet among children aged 6–23 months in Jig-Jiga town, Somali region Eastern Ethiopia, 2022 (n = 536)

Variables	Frequency	Percent
Age of mothers		
15–24 25–34 35–44 >=45	99 293 125 19	18.5 54.7 23.3 3.5
Marital status of mothers		
Married Divorced Widowed/Separated	502 19 15	93.7 3.5 2.8
Ethnicity of mothers		
Somali Others	417 119	77.8 22.2
Education of the mothers		
Cannot read and write Primary level Secondary and above	81 196 259	15.1 36.6 48.3
Education of the father		
Cannot read and write Primary level Secondary and above	17 104 415	3.2 19.4 77.4
Age of the child		
6–11 12–17 18–23	253 159 124	47.2 29.7 23.1

**Fig. 2:**
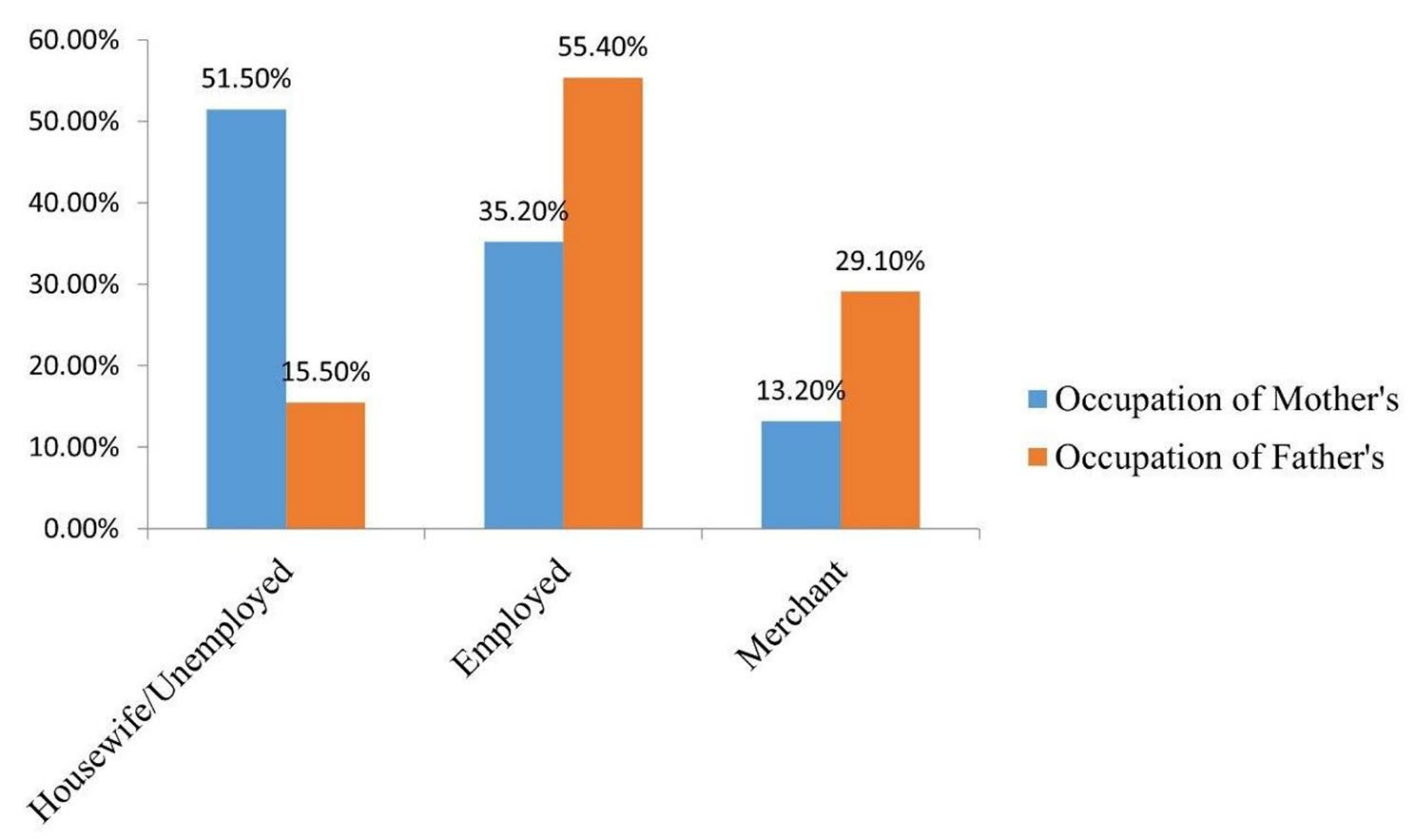
Mothers and Fathers of Children’s Occupational Status in the Study of Minimum Acceptable Diet among Children Aged 6–23 Months in Jig-Jiga Town, Somali Region, Eastern Ethiopia, 2022

Regarding the occupation of parents, half (26, or 51%) of the mothers were housewives or unemployed, and 297, or 55.4%, of the father’s occupation was government employment (Fig. 2).

### Household-related factors

The majority (434, 81%) of the households were headed by a husband. Concerning household decision-making, most—401 (74.8%) of the households—made decisions jointly. About 421 (78.5%) of the under-five children in the household had more than one child. More than half of the household members (340, or 63.5%) were aged 1–4. Moreover, most (336, or 62.7%) of the participant’s average monthly income was between 5,000 and 10,000 Ethiopian Birr (Table 2).

**Table 2:** Household characteristics of the study participant in the study of minimum acceptable diet among children aged 6–23 months in Jig-Jiga town, Somali region, Eastern Ethiopia, 2022 (n = 536)

Variables	Frequency	Percent
Does your husband have other wives?		
Yes No	131 405	24.4 75.6
No of under five children in the house hold		
1 child 2 and more child	421 115	78.5 21.5
Head of the household		
Husband Wife	434 102	81.0 19.0
Household decision-making		
Mother involved Mother not involved	401 135	74.8 25.2
Family size		
1–4 Families 5 and above	340 196	63.5 36.5
Average Monthly family income		
< 5000 5000-10,000 >=10,000	132 336 68	24.6 62.7 12.7

### Child-related characteristics

The majority (448, or 83.6%) of children were initiated on breast milk within one hour of delivery. Regarding the current status of breastfeeding, 335 (62.5%) children were breastfeeding. Regarding pre-lacteal feeding practice, 246 (45.6%) of the children were given pre-lacteal feeding, and 373 (69.6%) of the children were initiated on complementary feeding at 6 months. Concerning the frequency of feeding in 24 h, more than one-third (231, 43.1%) of mothers and caretakers fed their children three times, while 21 (3.9%), 139 (24.9%), and 145 (27.1%) of them fed their children one time, two times, and four or more times, respectively. The majority (515, or 96.1%) of children were vaccinated and had their vaccination cards; 415, or 84.1%, were not vaccinated. Children’s diarrheal and fever status in the last two weeks: 67 (12.5%) and 173 (32.3%) children have had diarrhea and fever, respectively, and the rest didn’t. The majority of children, 388 (74.2%), were not provided with child supplementation, but only 148 (27.6%) were (Table 3).

**Table 3:** Child characteristics of the study participants in the study of minimum acceptable diet among children aged 6–23 months in Jig-Jiga town, Somali region, Eastern Ethiopia, 2022 (n = 536)

Variables	Frequency	Percent
First breastfed for child after delivery		
Within 1 h After 1 h	448 88	83.6 16.4
Current status of breastfeeding		
Yes No	335 201	62.5 37.5
Child pre-lacteal feeding		
Yes No	246 290	45.9 54.1
When did you start complementary feeding		
Before 6 months At 6 months After 6 months	95 373 68	17.7 69.6 12.7
Frequency of feeding with in 24 h		
One times Two times Three times Four and above times	21 139 231 145	3.9 25.9 43.1 27.1
Child had diarrhea in the last two weeks		
Yes No	67 469	12.5 87.5

### Maternal health service utilization

In this study, the majority of 466 (86.9%) mothers were delivered at a health facility, 164 (30.6%) used mini-pills as contraceptives, and 295 (55%) did not use any contraceptives. Concerning the knowledge of mothers about the minimum acceptable diet, 391 (72.9%) of them knew minimum acceptable diet, and 266 (49.6%) of them received information from health extensions. Around 187 (34.9%) mothers received an extra meal, and 397 (74.1%) of them received supplementation during pregnancy or lactation. 311 people (58%) received IYCF counseling, according to the sources of information. The majority of 506 (94%) of the mothers had ANC flown up, 381 (71.1%) had postnatal follow-up after delivery, and 311 (58%) of them received IYCF counseling, while the rest of the mothers didn’t (Table 4).

**Table 4:** Maternal characteristics of the study participants in the study of MAD among children aged 6–23 months in Jig-Jiga town, Somali region, Eastern Ethiopia, 2022 (n = 536)

Variables	Frequency	Percent
Place of the delivery		
Home Health facility	28 508	5.2 94.8
Have you ever used contraceptive		
Yes No	241 295	45.0 55.0
Types of contraceptive		
Mini pills Others	164 77	30.6 14.4
Do you know minimum acceptable diet		
Yes No	391 145	72.9 27.1
What source of information regarding MAD		
Health extension Media Neighbors	266 42 83	49.6 7.8 15.5
Do you consume extra food during pregnancy/lactation		
Yes No	187 349	34.9 65.1
Received any supplements during pregnancy/lactation		
Yes No	397 139	74.1 25.9
Did you receive IYCF counselling		
Yes No	311 225	58.0 42.0

### The practice of a minimum acceptable diet

**Fig. 3:**
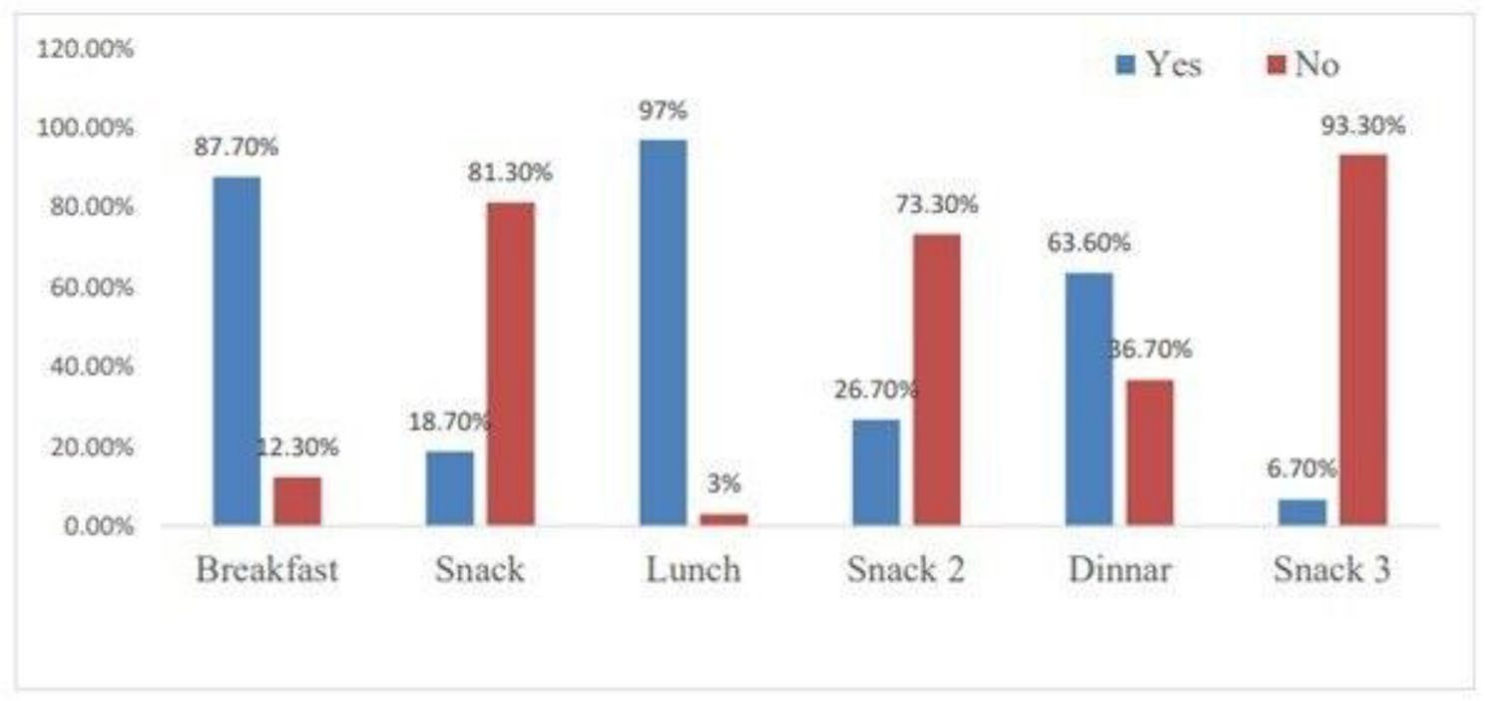
Minimum meal frequencies in the study of the minimum acceptable diet among children aged 6–23 months in Jig-Jiga town, Somali region, Eastern Ethiopia, 2022

The mainstream, 434 (81.7%) of children, were breastfed before the study. More than half, or 341 (68.5%) of children, met the minimum meal frequency (Fig. 3).

**Fig. 4:**
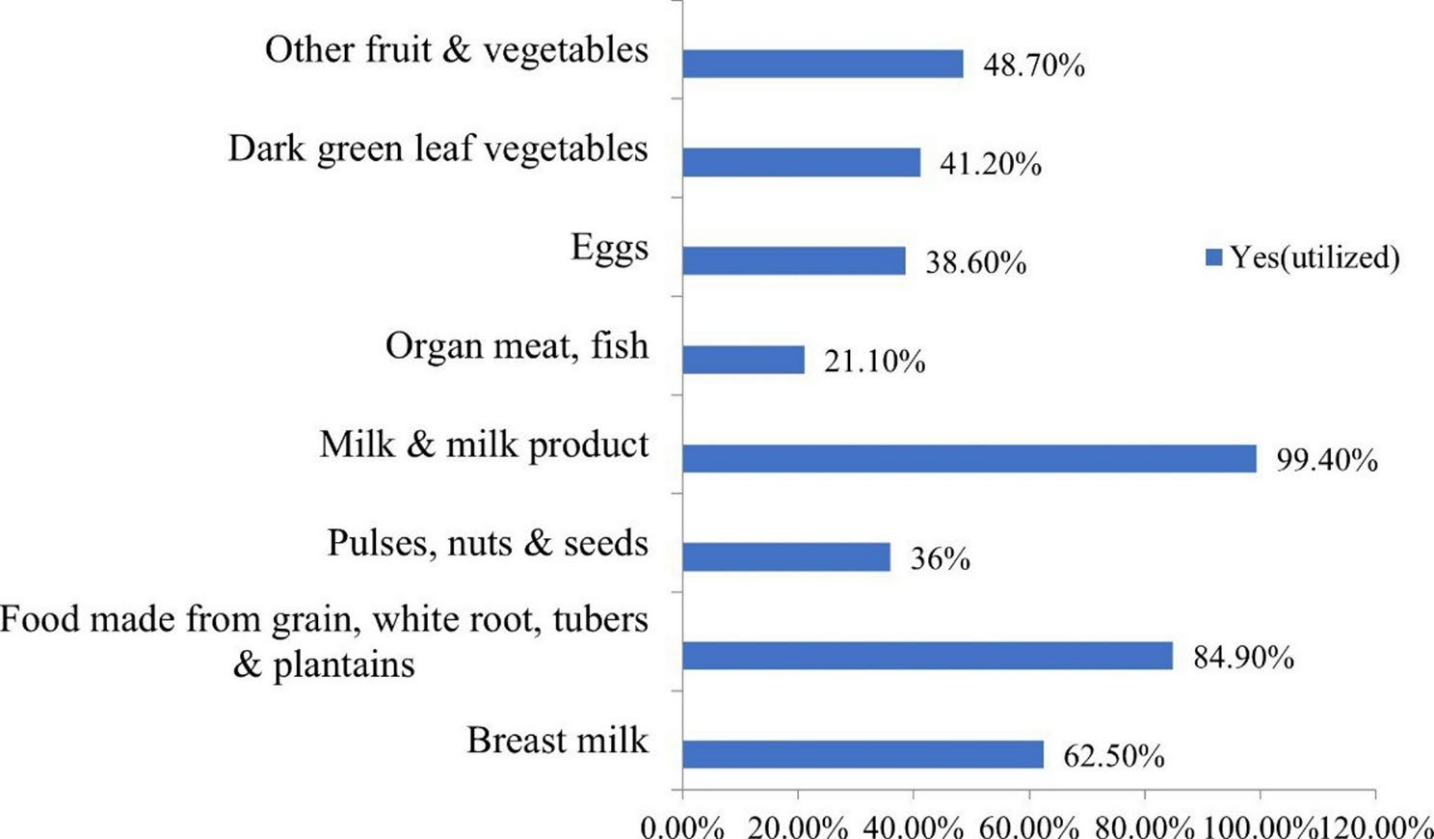
Minimum dietary diversity in the study of the minimum acceptable diet among children aged 6–23 months in Jig-Jiga town, Somali region, Eastern Ethiopia, 2022

In this study, 64.2% of respondents achieved the minimum dietary diversity. Milk and milk products were the most consumed food groups by 99.4% of study subjects, and organ meat and fish were the least consumed food groups by only 22.4% of children (Fig. 4).

**Fig. 5:**
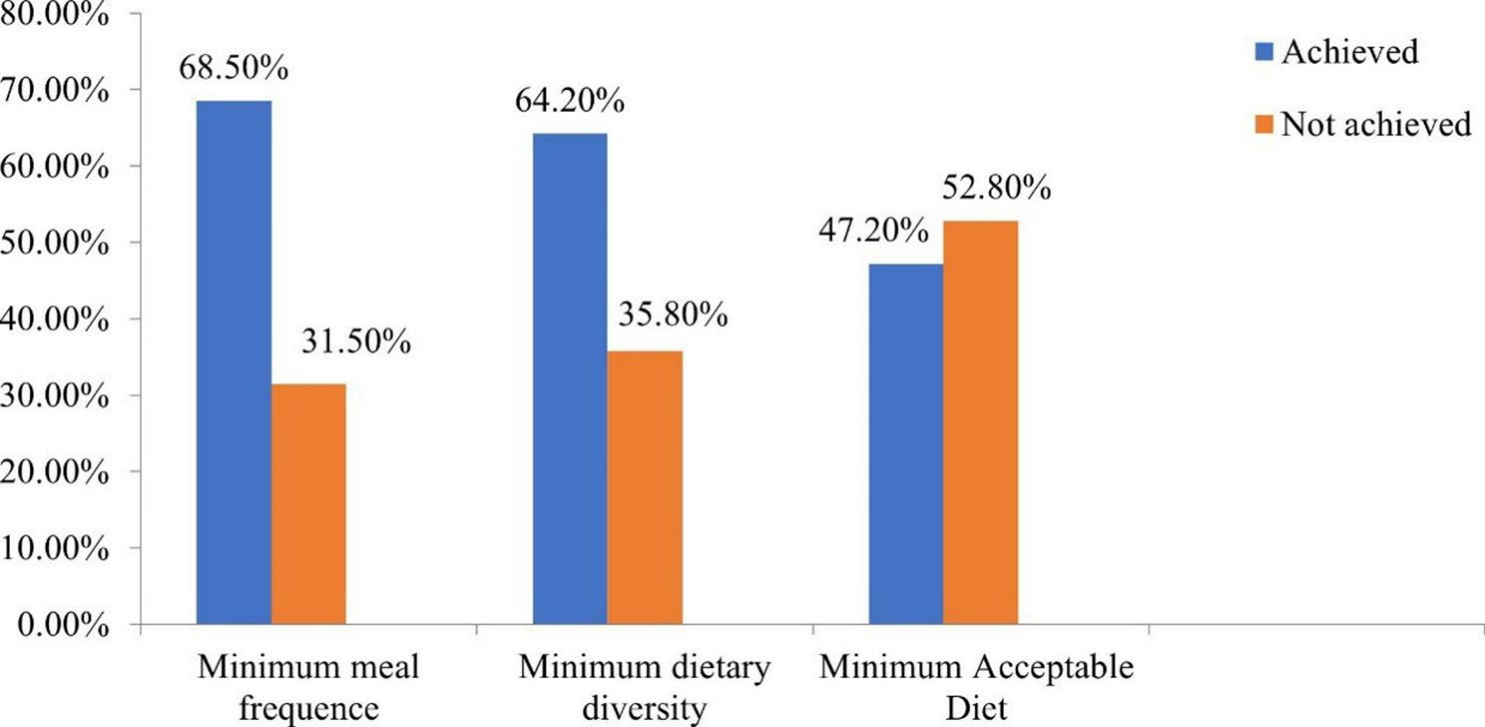
Minimum meal frequency, minimum dietary diversity, and minimum acceptable diet among children aged 6–23 months in Jig-Jiga town, Somali region, Eastern Ethiopia, 2022

About 47.2% of the children satisfied the recommended minimum acceptable diet standards. Six hundred and eight (68.5%) children received minimum meal frequency, and 64.2% of them received minimum dietary diversity. Four hundred twenty (64.6%) children started complementary feeding practice promptly, and 50.7% of them started complementary feeding at an appropriate time (Fig. 5).

### Factors associated with the minimum acceptable diet

To identify factors associated with a minimum acceptable diet, bivariate, and multivariate logistic regression were done. On the bivariate analysis, the minimum acceptable diet had statistically significant relationships with the occupation of the father, head of household, age of the child, number of under-five children, age of the mothers, current status of breastfeeding, ANC visit, and source of information regarding minimum acceptable diet, which had p < 0.25. In the multivariate analysis, five variables with a p-value less than 0.05 and 95% CI were confirmed: the occupation of the father, age of the child, age of the mother, under-five children, and source of information regarding the minimum acceptable diet were considered to be potential predictors of meeting the minimum acceptable diet. Children whose father’s occupation was government employment were 50% more likely to receive the minimum acceptable diet compared with children whose father’s occupation was unemployed or merchant; their AOR was 50%, 95% CI (0.3–0.8). Children aged 6–11 months were nearly four times more likely to meet the minimum acceptable diet (AOR = 3.6, 95% CI = 1.7–7.7), and those aged 12–17 months were nearly two times more likely (AOR = 2.1, 95% CI = 1.1–4.1) than children aged 18–23 months. Similarly, mothers aged 15–24 years were nearly eight times more likely to provide a minimum acceptable diet to their child than mothers aged 35–44 and > 45 years and mothers aged 25–34 were more than five times more likely to provide a minimum acceptable diet to their child than mothers aged 35–44 and > 45 years.

Mothers who had only one under-five child were two times (AOR = 2.1; 95% CI = 1.298–3.471) more likely to give a minimum acceptable diet than mothers who had two or more under-five children. Mothers who received minimum acceptable diet information from the media were 64% AOR = 0.16, 95% CI (0.061–0.433) more likely to provide the recommended minimum acceptable diet (Table 5).

**Table 5:** Bivariate and multivariate logistic regression of factors associated with a minimum acceptable diet among children aged 6–23 months in Jig-Jiga town, Eastern Ethiopia, 2022 (n = 536)

Variables category	Minimum acceptable diet		COR (95% CI)	AOR (95% CI)
	Achieved	Not achieved		
Occupation of the father				
Unemployed Government employed Merchant	46 142 65	37 155 91	1.74(0.336–0.983) 1.28 (0.527–1.153) 1	1.604(0.280–1.303 0.506(0.305–0.840)* 1
Head of the household				
Husband Wife	195 58	239 44	0.62 (1.046–2.496 1	0.712(0.371–1.366) 1
Age of the child				
6–11 12–17 18–23	86 79 88	167 80 36	1 4.75(2.976–7.572) 1.92(1.506–4.069)	**1** 3.654(1.712–7.796)* 2.150(1.118–4.137)*
Under five children				
1 2 and above	215 38	206 77	2.11(1.372–3.261) 1	2.123(1.298–3.471)* **1**
Age of mothers				
15–24 25–34 35–44 >=45	59 195 80 15	40 98 45 4	0.39(3.047–32.815) 0.53(1.403–13.35) 0.47(0.784–7.971) 1	7.68(1.547–38.146)* 5.56(1.174–26.325)* 3.493(0.744–16.397) 1
ANC visited				
Yes No	234 19	272 11	0.50(0.936–4.305) 1	0.703(0.228–2.174)1
Place of delivery				
Home Health facility	18 215	10 251	1 2.101(0.950–4.650)	1 0.957(0.452–2.024)
Source of information on MAD				
Health Extension Media Neighbors	116 24 33	150 18 50	1.17(1.059–2.392) 2.02(0.461–1.846) 1	0.562(0.303–1.042) 0.163(0.061–0.433)* 1

## Discussion

Attaining a minimum acceptable diet among children aged 6–23 months is one of the strategies currently used to break the intergenerational cycle of malnutrition. The proportion of children who met the minimum acceptable diet was only 47.2%. This study finding is higher compared to studies conducted in Nepal at 26.5% [33], Tanzania at 9% [34], Pakistan at 7.9% [35], Mareka district, Debre-Berhan town, Amhara region at 31.6% [3], Golina district, Afar region at 19.4%; [36], and the slums of Oromia zones at 29% [37]. This study’s findings are well-suited with studies done in southern Ethiopia (35.5%) [30]. However, this is lower than studies conducted in Addis Ababa (74.6%, [38]. This might be due to the mother’s education status, media exposure, and starting complementary feeding at the right time. In addition to this, the difference may be due to differences in study settings, socio-demographic, and economic characteristics of the study participants, as well as sampling size, study area, and period.

The findings of this study shown that the proportion of children who achieved minimum dietary diversity is higher than in studies conducted in Dembecha, North West Ethiopia (9.8%) [39], and North Shoa, Oromia Region, 45.5% [13]. These differences might be due to differences in the study area, study period, and educational status of mothers who are of reproductive age. This difference may also be due to cultural and economic variation among these populations.

The minimum meal frequency of this study was greater than studies done in Ethiopia, such as Dembecha, North West, 63% [39], Dangila Town, 50.4% [16], North Shoa, Oromia Region, 33% [13]. However, it was lower than studies conducted in Dabat District (72.2%, [40], Addis-Ababa (90.6%, [38] Filipino (93.5%, [18]. This might be due to differences in the study area, study period, and nutrition education exposure among populations.

Children aged 12–17 and 18–23 months were three and two times more likely, respectively, to meet the minimum acceptable diet than children aged 6–11 months. This study is compatible with similar studies conducted in Ghana [41], Pakistan [35], Mareka district, southern Ethiopia [30], and Debre-Berhan town [3]. This might be due to starting complementary feeding with either only milk or cereal products or with other types of food.

Children whose fathers were government employees were 50% more likely to meet the minimum acceptable diet than children whose fathers were unemployed. This indicates that government-employed fathers can help and give money to mothers who have children aged 6–23 months to purchase foods that increase the availability of some foods, like eggs, fruit, and vegetables. It could be due to increased awareness among government-employed fathers. Studies conducted in Mareka District [30], Addis-Ababa [38], and EDHS 2016 [20] were not supported in this study.

In this study, children whose mothers were exposed to media or got sources of information regarding the minimum acceptable diet and its factors throughout the media were 64% more likely to meet the minimum acceptable diet than those children of mothers who didn’t get such information throughout the media. This finding was similar to studies conducted in the North Shoa and Oromia regions [13], and the multi-level analysis report of EDHS 2016 [20]. This could be because media promotion enhances timely, adequate, and safe dietary practices. Moreover, the Ethiopian Ministry of Health and its partners promote child-feeding practices through radio and television. It increases the mother’s awareness of feeding a minimum acceptable diet to their child [26]. Also, this might be because mothers who have been exposed to the media have had better opportunities to access information on appropriate child-feeding practices.

This study also found that children born to mothers aged 15–24 years and 25–34 years were seven and five times more likely to receive the recommended minimum acceptable diet than children born to mothers aged 35–44 years and > 45 years, respectively. This finding was similar to one from a study conducted in Dabat District, North West Ethiopia [40], and Addis Ababa [38]. This may be related to the fact that mothers in this age group are highly productive and have better information about child-feeding practices.

### Strengths and limitations of the study

This study has a relatively large sample size, so generalizations are possible. This study shares all the limitations of a cross-sectional study design. Since minimum dietary diversity and meal frequency data were collected in the previous 24 h, it exposes a potential for recall bias among the respondents.

## Conclusion

This study showed that the prevalence of a minimum acceptable diet was low. Factors associated with a minimum acceptable diet included the father’s occupation, the child’s age, the mother’s age, having one under-five child, and the media as a source of information. Therefore, advocacy on nutritional behavioral change communication and strengthening nutritional education in infant and young child feeding are required to improve the provision of a minimum acceptable diet.

## Data Availability

The data that support the findings of this study are available from the corresponding author. No restrictions apply to the availability of these data and are publicly available. Publisher and Editor can request any data they need and be available from the corresponding author upon reasonable request at any time in need during the review process.

## Abbreviations

AHR: Adjusted hazard ratio
ANC: Anti-natal care
CHR: Cumulative hazard ratio
CI: confidence interval
EBF: exclusive breastfeeding
IYCF: Infant and young child feeding
MAD: minimum acceptable diet
MDD: Minimum dietary diversity
MMF: minimum meal frequency
ORS: oral rehydration system
PNC: Post-natal care
TFR: Total fertility rate
SPSS: social package for statistical software
UNICEF: United Nation International Child Emergency Fund
WHO: World health organization

## References

[1] World Health Organization. Essential nutrition actions: improving maternal, newborn, infant and young child health and nutrition.2013. 25473713

[2] Gillespie S, Menon P, Kennedy AL (2015). Scaling up impact on nutrition: what will it take?. Adv Nutr.

[3] Molla A, Egata G, Getacher L, Kebede B, Sayih A, Arega M (2021). Minimum acceptable diet and associated factors among infants and young children aged 6–23 months in Amhara region, Central Ethiopia: a community-based cross-sectional study. BMJ open.

[4] World Health Organization. “Indicators for assessing infant and young child feeding practices: part 2: measurement.“. (2010).

[5] Keeley B, Little C, Zuehlke E. The State of the World’s Children 2019: Children, Food, and Nutrition–Growing Well in a Changing World. UNICEF. 2019.

[6] Nkoka O, Mhone TG, Ntenda PA (2018). Factors associated with complementary feeding practices among children aged 6–23 mo in Malawi: an analysis of the demographic and Health Survey 2015–2016. Int health.

[7] UNICEF, UNFPA. Trends in maternal mortality 2000 to 2017: estimates by WHO. World Bank Group and the United Nations Population Division; 2019.

[8] Akombi BJ, Agho KE, Hall JJ, Wali N, Renzaho AM, Merom D (2017). Stunting, wasting and underweight in sub-saharan Africa: a systematic review. Int J Environ Res Public Health.

[9] Huffman SL, Schofield D (2011). Consequences of malnutrition in early life and strategies to improve maternal and child diets through targeted fortified products. Matern Child Nutr.

[10] UNICEF and WHO (2007). Indicators for assessing infant and young child feeding practices.

[11] White J, Mason J (2012). Assessing the impact on child nutrition of the Ethiopia Community-based Nutrition Program.

[12] Abera K (2012). Infant and young child feeding practices among mothers living in Harar, Ethiopia. Harar Bull Health Sci.

[13] Gizaw G, Tesfaye G (2019). Minimum acceptable diet and factor associated with it among infant and young children aged 6–23 months in north Shoa, Oromia region, Ethiopia. Int J Homeopathy Nat Med.

[14] Molla M, Ejigu T, Nega G. Complementary feeding practice and associated factors among mothers having children 6–23 months of age, Lasta District, Amhara region, Northeast Ethiopia. Advances in Public Health. 2017;2017.

[15] Beyene M, Worku AG, Wassie MM (2015). Dietary diversity, meal frequency and associated factors among infant and young children in Northwest Ethiopia: a cross-sectional study. BMC Public Health.

[16] Black RE, Allen LH, Bhutta ZA, Caulfield LE, De Onis M, Ezzati M (2008). Maternal and child undernutrition: global and regional exposures and health consequences. The Lancet.

[17] Khanal V, Sauer K, Zhao Y (2013). Determinants of complementary feeding practices among nepalese children aged 6–23 months: findings from demographic and health survey 2011. BMC Pediatr.

[18] Guirindola MO, Maniego MLV, Silvestre CJ, Acuin CCS (2018). Determinants of meeting the minimum acceptable diet among filipino children aged 6–23 months. Philipp J Sci.

[19] Central Statistical Agency (CSA) [Ethiopia] and ICF (2016). Ethiopia Demographic and Health Survey 2016.

[20] Tassew AA, Tekle DY, Belachew AB, Adhena BM (2019). Factors affecting feeding 6–23 months age children according to minimum acceptable diet in Ethiopia: a multilevel analysis of the Ethiopian Demographic Health Survey. PLoS ONE.

[21] Abdilahi MA, Nur AM, Jibril AD (2020). Prevalence of Acute Malnutrition and Associated factors among under-five children in Gursum District, Somali Region, Ethiopia. Science.

[22] Andualem A, Edmealem A, Tegegne B, Tilahun L, Damtie Y. Timely initiation of complementary feeding and associated factors among mothers of children aged 6–24 months in Dessie Referral Hospital, Northeast Ethiopia, 2019. Journal of Nutrition and Metabolism. 2020;2020. 10.1155/2020/6756202 PMC7803225 33489365

[23] Addis Ababa. Ethiopia. Abstract available from https://wfpha convex com/wfpha/2012/webprogram/Paper10587 HTML. 2005.

[24] World Health Organization (2010). Indicators for assessing infant and young child feeding Practices Country Profiles.

[25] World Health Organization. Global Nutrition Monitoring Framework: operational guidance for tracking progress in meeting targets for 2025.

[26] Birie B, Kassa A, Kebede E, Terefe B (2021). Minimum acceptable diet practice and its associated factors among children aged 6–23 months in rural communities of Goncha district, north West Ethiopia. BMC Nutr.

[27] WHO U, USAID A, AED U (2008). Indicators for assessing infant and young child feeding practices.

[28] World Health Organization. Reproductive Health. Medical eligibility criteria for contraceptive use. World Health Organization; 2010.

[29] Zisovska E. WHO Technical Consultation on Postpartum and Postnatal Care. 2011. 26269861

[30] Feleke FW, Mulaw GF (2020). Minimum acceptable diet and its predictors among children aged 6–23 months in Mareka District, southern Ethiopia: a community-based cross-sectional study. Int J Child Health Nutr.

[31] Yesuf NN, Mekonnen EG, Takele WW (2021). Minimum dietary diversity and associated factors among young infants and children living in the most productive area of Amhara region, Addis Zemen town: a community-based cross-sectional study. Int J Afr Nurs Sci.

[32] Framework WGNM (2017). Operational guidance for tracking progress in meeting targets for 2025.

[33] Kundu RN, Hossain MG, Haque MA, Biswas S, Huq MM, Pasa MK (2022). Factor associated with anthropometric failure among under-five Bengali children: a comparative study between Bangladesh and India. PLoS ONE.

[34] Victor R, Baines SK, Agho KE, Dibley MJ (2014). Factors associated with inappropriate complementary feeding practices among children aged 6–23 months in T anzania. Matern Child Nutr.

[35] Na M, Aguayo VM, Arimond M, Stewart CP (2017). Risk factors of poor complementary feeding practices in pakistani children aged 6–23 months: a multilevel analysis of the demographic and Health Survey 2012–2013. Matern Child Nutr.

[36] Fentaw Mulaw G, Wassie Feleke F, Adane Masresha S. Maternal characteristics are associated with child dietary diversity score, in Golina District, Northeast Ethiopia: a community-based cross-sectional study. Journal of Nutrition and Metabolism. 2020;2020. 10.1155/2020/6702036 PMC7528112 33029394

[37] Issaka AI, Agho KE, Page AN, Burns PL, Stevens GJ, Dibley MJ (2015). Determinants of suboptimal complementary feeding practices among children aged 6–23 months in four anglophone W est African countries. Matern Child Nutr.

[38] Abebe H, Gashu M, Kebede A, Abata H, Yeshaneh A, Workye H (2021). Minimum acceptable diet and associated factors among children aged 6–23 months in Ethiopia. Ital J Pediatr.

[39] Mulat E, Alem G, Woyraw W, Temesgen H (2019). Uptake of minimum acceptable diet among children aged 6–23 months in orthodox religion followers during fasting season in a rural area, DEMBECHA, north West Ethiopia. BMC Nutr.

[40] Belew AK, Ali BM, Abebe Z, Dachew BA (2017). Dietary diversity and meal frequency among infant and young children: a community-based study. Ital J Pediatr.

[41] Issaka AI, Agho KE, Burns P, Page A, Dibley MJ (2015). Determinants of inadequate complementary feeding practices among children aged 6–23 months in Ghana. Public Health Nutr.

